# The identification of novel gene mutations for degenerative lumbar spinal stenosis using whole-exome sequencing in a Chinese cohort

**DOI:** 10.1186/s12920-021-00981-4

**Published:** 2021-05-21

**Authors:** Xin Jiang, Dong Chen

**Affiliations:** Department of Orthopaedics, China-Japan Friendship Hospital, No. 2 Yinghuayuan Dongjie, Chaoyang District, Beijing, 100029 China

**Keywords:** Degenerative lumbar spinal stenosis, Whole-exome sequencing, Susceptible genes, Single nucleotide polymorphisms, Gene mutations

## Abstract

**Background:**

Degenerative lumbar spinal stenosis (DLSS) is a common lumbar disease that requires surgery. Previous studies have indicated that genetic mutations are implicated in DLSS. However, studies on specific gene mutations are scarce. Whole-exome sequencing (WES) is a valuable research tool that identifies disease-causing genes and could become an effective strategy to investigate DLSS pathogenesis.

**Methods:**

From January 2016 to December 2017, we recruited 50 unrelated patients with symptoms consistent with DLSS and 25 unrelated healthy controls. We conducted WES and exome data analysis to identify susceptible genes. Allele mutations firstly identified potential DLSS variants in controls to the patients’ group. We conducted a site-based association analysis to identify pathogenic variants using PolyPhen2, SIFT, Mutation Taster, Combined Annotation Dependent Depletion, and Phenolyzer algorithms. Potential variants were further confirmed using manual curation and validated using Sanger sequencing.

**Results:**

In this cohort, the major classification variant was missense_mutation, the major variant type was single nucleotide polymorphism (SNP), and the major single nucleotide variation was C > T. Multiple SNPs in 34 genes were identified when filtered allele mutations in controls to retain only patient mutations. Pathway enrichment analyses revealed that mutated genes were mainly enriched for immune response-related signaling pathways. Using the Novegene database, site-based associations revealed several novel variants, including *HLA-DRB1*, *PARK2, ACTR8, AOAH, BCORL1, MKRN2, NRG4, NUP205* genes*,* etc*.,* were DLSS related.

**Conclusions:**

Our study revealed that deleterious mutations in several genes might contribute to DLSS etiology. By screening and confirming susceptibility genes using WES, we provided more information on disease pathogenesis. Further WES studies incorporating larger DLSS patient cohorts are required to comprehend the genetic landscape of DLSS pathophysiology fully.

**Supplementary Information:**

The online version contains supplementary material available at 10.1186/s12920-021-00981-4.

## Background

Lumbar spinal stenosis (LSS) is characterized by narrowing the lumbar spinal canal, lateral recesses, or intervertebral foramina [1, 2]. The condition may be congenital, acquired, or degenerative [3–5]. Congenital stenosis is rare and typically caused by achondroplasia and hypochondroplasia. Degenerative lumbar spinal stenosis (DLSS) is usually attributed to degenerative changes in intervertebral discs, facet joints, and ligamentum flavum, leading to spondylolisthesis. DLSS typically affects individuals over 50 years old and globally is one of the most common spinal conditions requiring surgery. Neurogenic claudication is the classical clinical manifestation associated with LSS. It causes diminished function and impairs quality of life [6–9].

DLSS etiology and patho-mechanisms remain unclear, but previous studies implicate genetic factors in DLSS. A Finnish study identified a splice site variant of *COL9A2* associated with the condition [10]. Seung-Jae Hyun reported a *COL9A2* haplotype (HAP2) was significantly associated with DLSS in the Korean population, whereas another haplotype (HAP4) potentially exerted a protective role against DLSS development [11]. Currently, there is limited information on susceptibility gene mutations for DLSS. Therefore, the identification of pathogenic genes will provide novel therapeutic strategies for DLSS treatment.

Whole-exome sequencing (WES) is a cost-effective, reproducible, and robust approach for the sensitive and specific identification of variants causing protein-coding changes in the human genome. Previous genetic studies were limited to identifying one or more candidate genes, whereas WES approaches can potentially identify and screen hundreds of thousands of single nucleotide polymorphisms (SNPs) [12]. For populations with pathogenic stenosis gene mutations, preventative measures, such as lifestyle modifications and better stenosis monitoring, may be applicable. In this study, we analyzed WES data from 50 DLSS patients and 25 healthy controls to identify pathogenic genes, used Sanger DNA sequencing to validate variants identified by WES, and analyzed SNPs using bioinformatics analysis. We identified several pathogenic gene variants associated with DLSS in our cohort.

## Methods

### Study population

We recruited 50 consecutive, unrelated Chinese patients with symptoms consistent with DLSS and 25 unrelated healthy controls between January 2016 and December 2017 from the Orthopedic Department at the China-Japan Friendship Hospital. The ethics committee approved this study of the Hospital Institutional Review Board. All participants provided written informed consent to use samples and clinical information. All patients underwent clinical and radiological examinations to confirm the degenerative nature of their stenosis. DLSS patients met the following three criteria: (1) DLSS diagnosis by magnetic resonance imaging (MRI); (2) conservative treatment and monitoring for > three months by spinal surgeons, and (3) a history of typical LSS symptoms, consisting of self-reported intermittent neurogenic claudication, stenotic symptoms extending to the lower extremities upon extension of the lumbar spine, or numbness or weakness of the lower extremities. The study excluded patients with neurological disorders, spinal fractures, spondylolisthesis, spinal tumors, trauma, and infectious diseases. We enrolled 25 biologically unrelated healthy individuals with similar ethnic backgrounds as the control group.

### WES

Genome DNA was extracted from whole blood using the TIANamp Blood DNA kit (TIANGEN BIOTECH, Beijing, China). We performed exome capture using the Agilent SureSelect Human All Exon V6 kit (Agilent Technologies, Santa Clara, CA, USA) as per the manufacturer’s instructions. WES was performed using the Illumina HiSeq Xten platform. We processed sequencing-derived raw image files using Illumina base-calling software with default parameters and generated sequence data for each individual as paired-end reads or raw data. We conducted studies according to the manufacturer’s protocol. One sample was loaded per flow cell lane to generate a minimum 10 × read depth across ~ 96% target regions.

### Exome data analysis

**Fig. 1:**
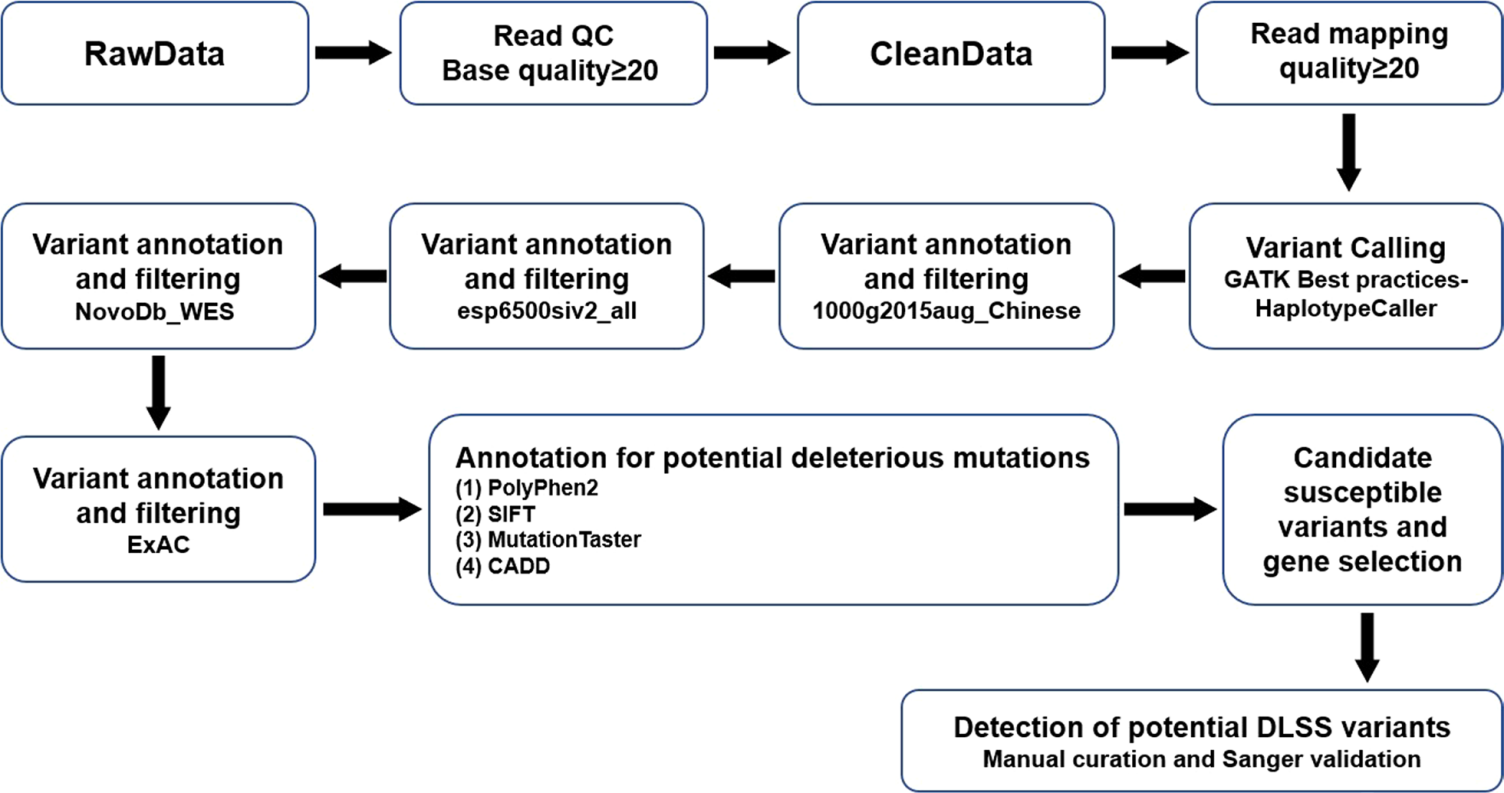
Bioinformatics filtering pipeline showing how raw data were processed

The bioinformatics analysis used raw sequencing data from the Illumina pipeline (Fig. 1). We processed and filtered the raw data and discarded low-quality reads based on the following criteria: (1) reads containing an abnormal sequencing adapter, (2) reads with a low-quality base ratio (base quality less than or equal to 5 that was more than 50%, and (3) reads with an unknown base (“N” base) ratio > 10%. Variants in exon or alternative splicing regions were retained. We compared the variant frequency using the 1000G SNP database (http://www.1000genomes.org/). Exome Aggregation Consortium (ExAC) (http://exac.broadinstitute.org) analysis for potential deleterious mutations was performed using different algorithms; PolyPhen2 (http://genetics.bwh.harvard.edu/pph2/) [13], SIFT (http://sif.jcvi.org/) [14], Mutation Taster (http://www.mutationtaster.org/) [15], and Combined Annotation Dependent Depletion (CADD). We used the identification of rare combined with at least two of these four algorithms to prioritize potential pathogenic variants. We used R software (http://www.bioconductor.org/packages/release/bioc/html/maftools.html) for cluster analyses to determine statistical significance in genotype and allele frequencies between patients and controls (false discovery rate (FDR) < 0.05). Phenolyzer (Phenotype Based Gene Analyzer, http://phenolyzer.wglab.org/) was used to identify genes based on user-specific disease/phenotype terms.

### Read mapping to reference sequences

Single nucleotide variants (SNVs) in the 75 samples were generated by comparing valid sequencing data with the human reference genome (UCSC Genome Browser hg19) using Burrows–Wheeler Aligner software to derive primary mapping results. Then, we sorted the aligned data using SAM tools to select the best mapping positions. Duplicated reads were marked by Picard (http://sourceforge.net/ projects/picard/) for use in the next analysis.

Also, we conducted functional annotations to determine genetic variations associated with DLSS. Firstly, we annotated all variants using ANNOVAR software. Then, normal population variant databases, including 1000g2015aug_all, 1000g2015aug_Chinese, esp6500siv2_all, NovoDb_WES_SNP, and ExAC databases, were screened to exclude common variations occurring with no more than 1% minor allele frequency.

### Candidate variants and gene selection

SIFT, PolyPhen-2, and MutationTaster analyses were performed to predict whether amino acid substitutions affected protein function. A mutation was selected as a candidate variant if one of the three software programs showed it was pathological.

### Gene function enrichment analysis

The Toppgene suite was used for gene enrichment analysis, and candidate gene prioritization was based on functional annotation and a protein interaction network (https://toppgene.cchmc.org/). We used a web-based portal, Metascape (http://metascape.org/), to perform Gene Ontology (GO) enrichment analysis. Gene Set Enrichment Analysis (GSEA) software (version 3.0) with a “c2.cp.kegg.v6.1symbols.gmt” gene set database was used to perform GSEA (http://www.broadinstitute.org/gsea/index.jsp) to identify significantly enriched or depleted genes that showed statistically significant and concordant differences between two given clusters.

### Statistical analysis

We performed a Fisher’s exact test to evaluate associations between rare variants and disease phenotype (case/control data) using the Benjamini and-Hochberg method, a type of FDR test used for multiple hypothesis testing to correct for multiple comparisons.

## Results

### Patient cohort

The study included 21 male and 29 female patients. Patients had an average age of 52.4 years, ranging from 46 to 70 years. Also, 10 male and 15 female controls were included, with an average age of 52.4 years, ranging from 29 to 70 years. After Principal Component Analysis, three patients were deemed as outliers. Without these outliers, the remaining 72 participants were in a random distribution. The three outliers (bijinfang, bijinfen, bijinhua) had a close genetic relationship (Additional file 1: Fig. S1, Additional file 2: Fig. S2).

### General variant status of participants using WES

We performed WES on 75 DNA samples using the Illumina Hiseq Xen platform. To filter potential pathogenic variants, we focused on the identification of rare 1000G_EAS ≤ 0.005, based on the BGI database) and damaging variants predicted by at least two of four algorithms (i.e.,., SIFT, Polyphen2, Mutation Assessor, Mutation Taster, and CADD). Detrimental variants were classified as missense_mutation, frame_shift_del, nonsense_mutation, frame_shift_ins, splice_site, in_frame_ins, in_frame_del, nonstop_mutation. The basic variant status in the 75 samples is shown (Additional file 3: Fig. S3, Additional file 4: Fig. S4).

### DLSS-related variant identification from 72 participants

**Fig. 2:**
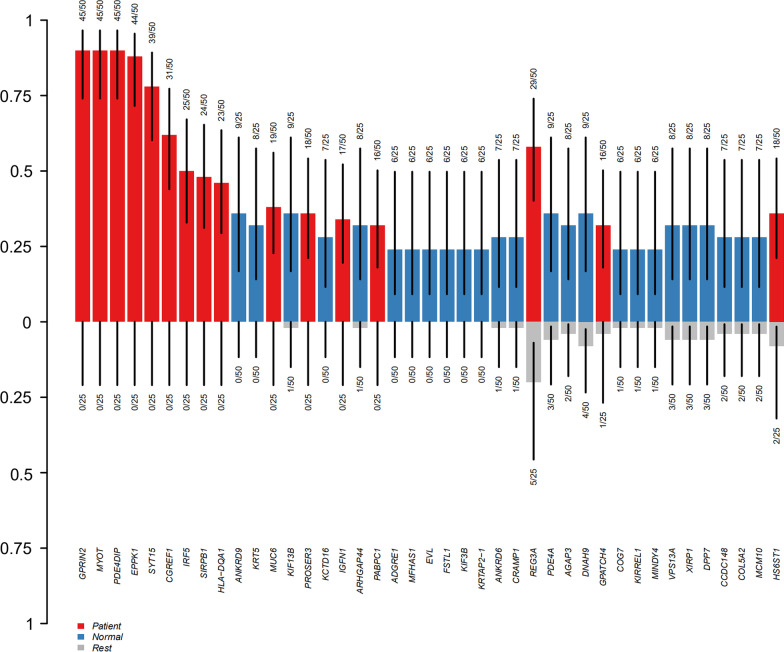
Candidate gene mutation frequencies in both groups

Genes related to DLSS were processed by cluster analysis in R software. We filtered allele mutations in controls to retain only mutations in patients and identified multiple SNPs in 34 genes (FDR < 0.05) (Fig. 2, Table 1).

**Table 1:** Candidate genes by clustering analysis by R software

GENE	Feature_1	Feature_2	N_of_Feature1	N_of_Feature2	FDR
*GPRIN2*	Patient	Normal	45 of 50	0 of 25	2.70983E−15
*MYOT*	Patient	Normal	45 of 50	0 of 25	2.70983E−15
*PDE4DIP*	Patient	Normal	45 of 50	0 of 25	2.70983E−15
*EPPK1*	Patient	Normal	44 of 50	0 of 25	1.40008E−14
*SYT15*	Patient	Normal	39 of 50	0 of 25	1.14246E−11
*CGREF1*	Patient	Normal	31 of 50	0 of 25	3.2011E−08
*IRF5*	Patient	Normal	25 of 50	0 of 25	3.3725E−06
*SIRPB1*	Patient	Normal	24 of 50	0 of 25	9.2172E−06
*HLA-DQA1*	Patient	Normal	23 of 50	0 of 25	1.06146E−05
*MUC6*	Patient	Normal	19 of 50	0 of 25	0.000141694
*PROSER3*	Patient	Normal	18 of 50	0 of 25	0.000315482
*IGFN1*	Patient	Normal	17 of 50	0 of 25	0.000375298
*PABPC1*	Patient	Normal	16 of 50	0 of 25	0.000728519
*KRT79*	Patient	Normal	9 of 50	0 of 25	0.02508378
*FLNA*	Patient	Normal	9 of 50	0 of 25	0.02508378
*PHRF1*	Patient	Normal	9 of 50	0 of 25	0.02508378
*ALS2CL*	Patient	Normal	10 of 50	0 of 25	0.025483837
*KNSTRN*	Patient	Normal	10 of 50	0 of 25	0.025483837
*COL6A5*	Patient	Normal	8 of 50	0 of 25	0.046170366
*TSHZ2*	Patient	Normal	8 of 50	0 of 25	0.046170366
*AADAC*	Patient	Normal	8 of 50	0 of 25	0.046170366
*ADAT1*	Patient	Normal	8 of 50	0 of 25	0.046170366
*CMA1*	Patient	Normal	8 of 50	0 of 25	0.046170366
*DEAF1*	Patient	Normal	8 of 50	0 of 25	0.046170366
*DLAT*	Patient	Normal	8 of 50	0 of 25	0.046170366
*DST*	Patient	Normal	8 of 50	0 of 25	0.046170366
*FLG*	Patient	Normal	8 of 50	0 of 25	0.046170366
*KIAA0556*	Patient	Normal	8 of 50	0 of 25	0.046170366
*LPA*	Patient	Normal	8 of 50	0 of 25	0.046170366
*P2RX5*	Patient	Normal	8 of 50	0 of 25	0.046170366
*PANK2*	Patient	Normal	8 of 50	0 of 25	0.046170366
*RASSF4*	Patient	Normal	8 of 50	0 of 25	0.046170366
*WARS*	Patient	Normal	8 of 50	0 of 25	0.046170366
*ZNF572*	Patient	Normal	8 of 50	0 of 25	0.046170366

Frequency of allele mutations were zero in controls and FDR < 0.05

**Fig. 3:**
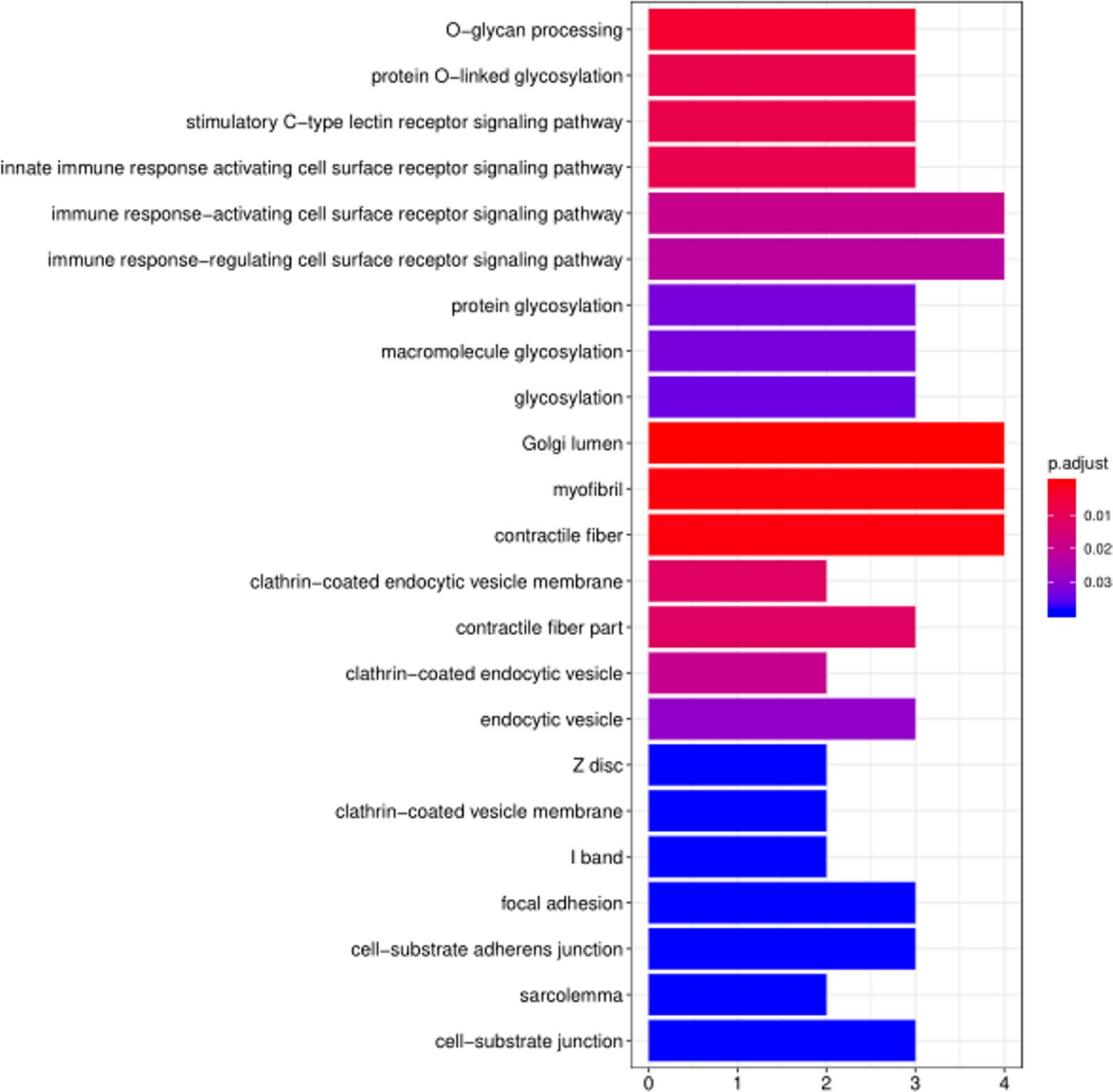
Enriched pathways possibly related to DLSS

We analyzed differences using GO and pathway analysis to differentiate specified characteristics between patients and controls (Fig. 3, Additional file 7: Table S1).

### DLSS-related variant identification using public databases

**Fig. 4:**
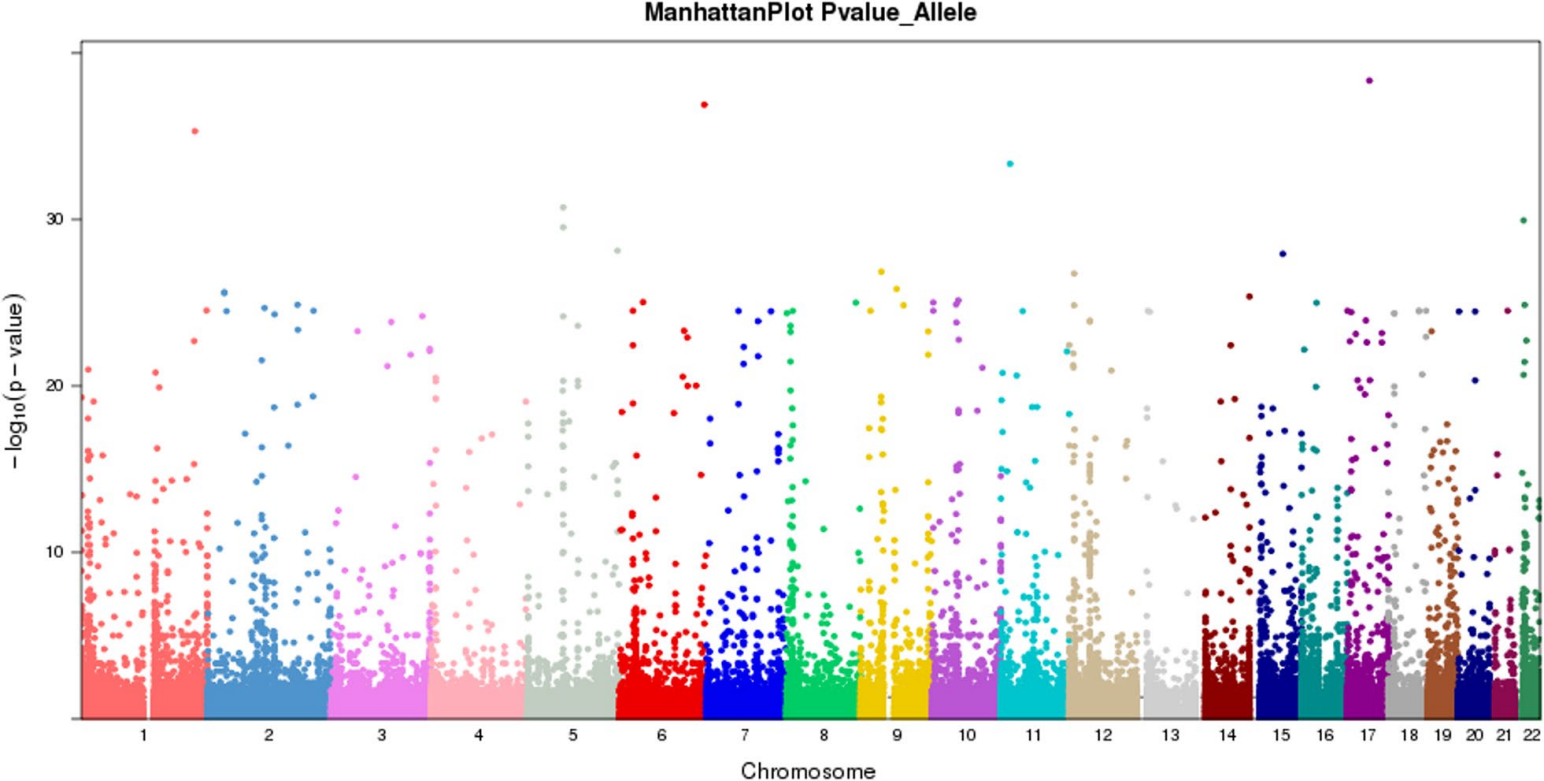
ManhattanPlot Genome-wide spread of base-10 log of *P* values. Each signal is a gene-based − log10 (*P* value)

The study used 50 disease and 2245 control cases (Novegene database) to perform site-based associations (Fig. 4). Forty-three (43) genes were filtered up to *P* value ≤ 0.001 and FDR_BH_Allele ≤ 0.01. After Toppgene analyses, the top two genes were *HLA-DRB1* and *PRKN*. Table 2 shows the top four SNPs related to lumbar disease. We also performed pathway and Phenolyzer analysis to discover genes related to lumbar disease. Genes related to DLSS (Phenolyzer score ≥ 0.01) are shown (Additional file 5: Fig. S5).

**Table 2:** Top four SNP filtered related to lumbar disc degeneration

Chromosome ID		Ref	Alt	Alt case	Alt control	Ref case	Ref control	*P* value	Function
6	rs113663708	G	T	14	199	78	4151	0.0001	exonic
6	rs201219028	G	A	29	724	63	3616	0.000602	splicing
6	rs9269955	G	C	40	1110	58	3328	0.000883	exonic; splicing
6	rs12190823	G	A	42	1179	50	3289	0.000109	intronic

After filtering data using 1000G_EAS ≤ 0.005 and damaging variants predicted by at least two of four algorithms (i.e., SIFT, Polyphen2, Mutation Assessor, Mutation Taster, and CADD), we identified 322 SNPs in 60 candidate genes, with *P* values ≤ 0.001. We also performed pathway and Phenolyzer analyses to discover genes related to lumbar disease. As “lumbar degeneration” was not a disease item, we inputted “lumbar disc degeneration” into Phenolyzer. Genes related to DLSS (Phenolyzer scores ≥ 0.01) are shown (Table 3 and Additional file 6: Fig. S6).

**Table 3:** Top 6 genes filtered from 60 genes by phenolyzer analysis

Gene	Alt_Case	Ref_Case	Alt_Control	Ref_Control	*P* value
*PLEC*	9	91	0	4490	7.69E−16
*SHC4*	5	95	31	4459	0.000979097
*NRG4*	3	97	4	4486	0.00033
*TNKS*	2	98	0	4490	0.00047
*DKK4*	2	98	0	4490	0.00047
*RHPN1*	2	98	0	4490	0.00047

Phenolyzer score ≥ 0.01

As there was a genetic relationship between bijingfang, bijinfen, and bijinhua, not all patients had a sporadic disease. We sought to determine SNPs not only in these three cases but also in the remaining 47. Six potential genes were identified associated with DLSS (Tables 4, 5).

**Table 4:** The screening procedures to find the six genes excluding family influence

			bijinfang
		bijinfang	bijinfen
	bijinfang	bijinfen	bijinhua
Nonsynonymous cSNP, splice site variatant or coding indel (NS/SS/I)	1060	657	87
NS/SS/I not dbSNP	133	76	30
NS/SS/I not 1000g2015aug_all	133	76	30
NS/SS/I not 1000g2015aug_Chinese	133	76	30
NS/SS/I not esp6500siv2_all	133	76	30
NS/SS/I not NovoDb_WES_SNP	113	63	19
NS/SS/I not ExAC	113	63	19
	29	15	6

**Table 5:** Six novel genes identified to have relations with DLSS with high possibility

Gene	47 Case		50 case	
	1000g2015aug_all < 0.01	ExAC_ALL < 0.01	1000g2015aug_all < 0.01	ExAC_ALL < 0.01
*ACTR8*	13	1	16	4
*AOAH*	13	10	16	13
*BCORL1*	3	3	6	6
*MKRN2*	3	3	6	6
*NRG4*	6	6	9	9
*NUP205*	2	2	5	5

## Discussion

Previous evidence has indicated that genetic factors may be implicated in DLSS; therefore, we performed WES on 50 patients and 25 controls to investigate disease contributing genes. We identified several novel candidate genes previously unconnected with DLSS.

Several studies have identified candidate genes associated with lumbar disc degeneration. Jason et al*.* performed a genome-wide association study and identified multiple SNPs suggesting a multifactorial basis for DLSS [16]. In other work, the *HLA-DRB1* genotype increased the risk of developing pain after surgery or lumbar disc herniation [17]. A *PARK2* gene variant was associated with lumbar disc degeneration by influencing overall *PARK2* methylation [18]*.* In this study, *HLA-DRB1* and *PARK2* were identified as susceptibility genes associated with a predisposition to DLSS. Lumbar disc degeneration causes intervertebral collapse, which may accelerate DLSS development, suggesting overlapping mechanisms exist between the two degenerative processes. Our genetic data partially agreed with previous studies; however, specific gene-related DLSS mechanisms require further investigation.

This study also identified *ACTR8, AOAH, BCORL1, MKRN2, NRG4,* and *NUP205* genes associated with DLSS. *ACTR8* has 14 exons, is located on chromosome 3p21.1, with mutations associated with lineage-specific expression in primates [19]. *AOAH* is found on chromosome 7p14.2 and encodes both light and heavy acyloxyacyl hydrolaseregion subunits. *AOAH* polymorphisms are reportedly associated with asthma, chronic rhinosinusitis, and bronchial hyperreactivity [20, 21]. Located on chromosome Xq26.1, *BCORL1* encodes a transcriptional corepressor that tethers promoter regions via DNA-binding proteins*.* Pathogenic *BCORL1* variants reportedly underlie a newly identified X-linked epigenetic syndrome [22]. *MKRN2* is located on chromosome 3p25.2 and encodes a putative E3 ubiquitin ligase containing several zinc finger domains. The gene is involved in inflammatory response regulation and is implicated in non-small-cell lung cancer [23, 24]. *NRG4* is located on chromosome 15q24.2 and is a member of the epidermal growth factor family of extracellular ligands, is highly expressed in adipose tissue, enriched in brown fat, and markedly increased during brown adipocyte differentiation [25]. *NUP205* on chromosome7q33 encodes a nucleoporin which is a subunit of the nuclear pore complex which functions in protein, RNA, and ribonucleoprotein particle active transport between the nucleus and cytoplasm. Mutations in *NUP205* are associated with steroid-resistant nephrotic syndrome [26].

Several other DLSS candidate genes were also identified in this study, including *GPRIN2*, *MYOT*, and *PDE4DIP*, etc. Several pathways were enriched using differentially expressed gene analysis between patients and controls. Until now, these genes were not associated with DLSS.

Our study had several limitations. Firstly, patient numbers (50) were largely inadequate for a genetic study. However, we had enrolled three family members. In terms of future work, the candidate genes identified here warrant further investigation. Functional studies should be conducted to determine how candidate gene molecular mechanisms and pathways impact DLSS development. However, our immediate remit is to increase cohort size to increase statistical power and identify more susceptible genes.

## Conclusions

We identified several candidate gene mutations potentially associated with DLSS in Chinese patients using WES for the first time. Further verification of our data may help develop molecular-based approaches to aid DLSS diagnosis and treatment.

## Supplementary Information

**Additional file 1: Fig. S1.** Principal Component Analysis of 75 samples.

**Additional file 2: Fig. S2.** Principal Component Analysis of 72 samples.

**Additional file 3: Fig. S3.** Basic variant status in the 75 cases.

**Additional file 4: Fig. S4.** Basic variant status in the 75 cases.

**Additional file 5: Fig. S5.** Forty-three genes identified by Phenolyzer analysis (Phenolyzer score ≥ 0.01).

**Additional file 6: Fig. S6.** Sixty genes identified by Phenolyzer analysis (Phenolyzer score ≥ 0.01).

**Additional file 7: Table. S1.** Pathways enriched possibly related to DLSS.

## Data Availability

The datasets generated and/or analysed during the current study are available in the [SRA] repository, [https://www.ncbi.nlm.nih.gov/bioproject/PRJNA728520/].

## Abbreviations

DLSS: Degenerative lumbar spinal stenosis
WES: Whole exome sequencing
GO: Gene Ontology
MRI: Magnetic resonance imaging
SNVs: Single nucleotide variants
BWA: Burrows–Wheeler Aligner
MAF: Minor allele frequency
