# Supplementary figures and images for: The identification of novel gene mutations for degenerative lumbar spinal stenosis using whole-exome sequencing in a Chinese cohort
Source: BMC Med Genomics. 2021 May 21;14:134. doi: 10.1186/s12920-021-00981-4 (PMC8138972; doi:10.1186/s12920-021-00981-4)

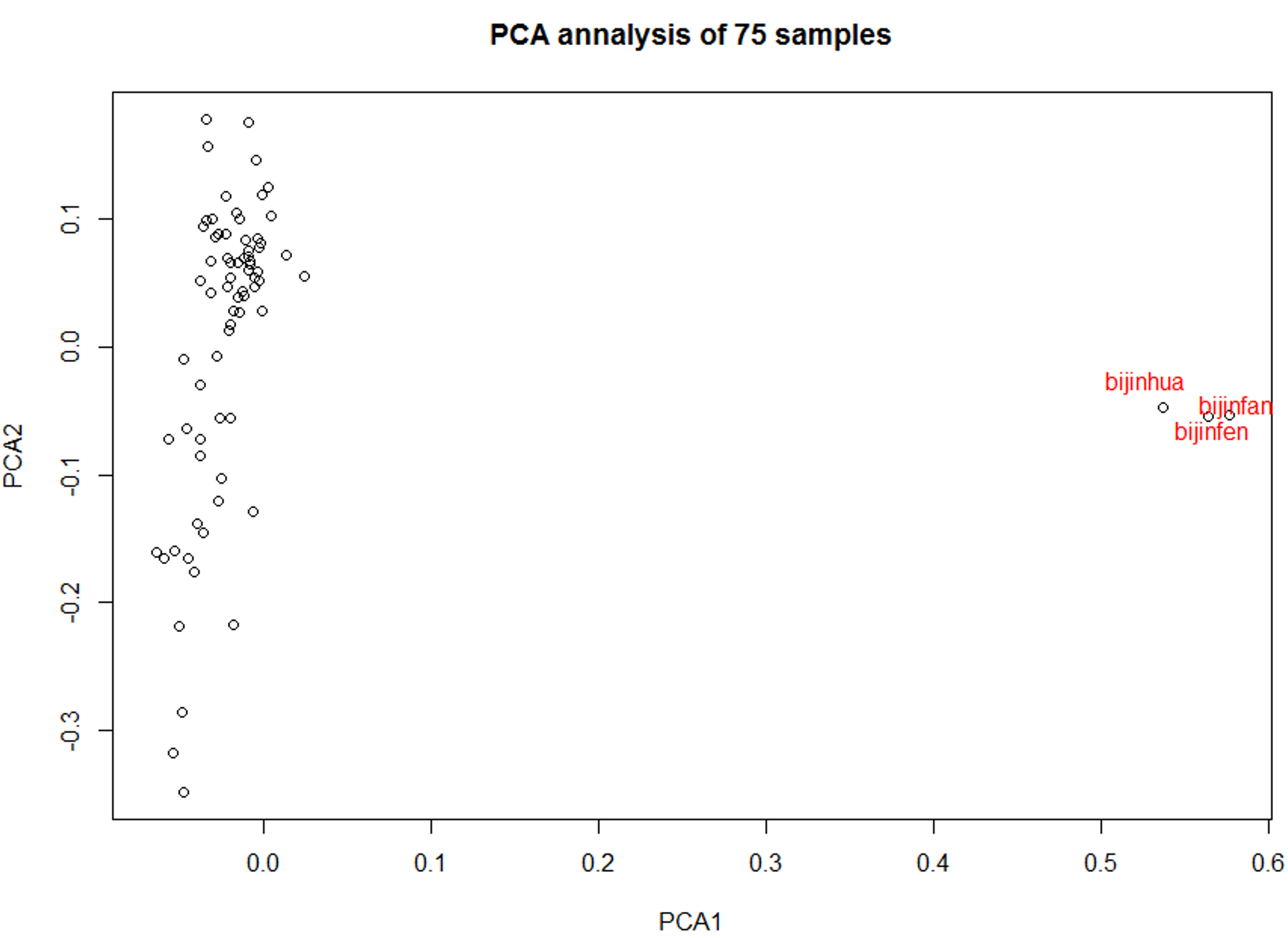

Supplement: Supplementary file 1 — Additional file 1: Fig. S1. Principal Component Analysis of 75 samples. [file 12920_2021_981_MOESM1_ESM.tif]

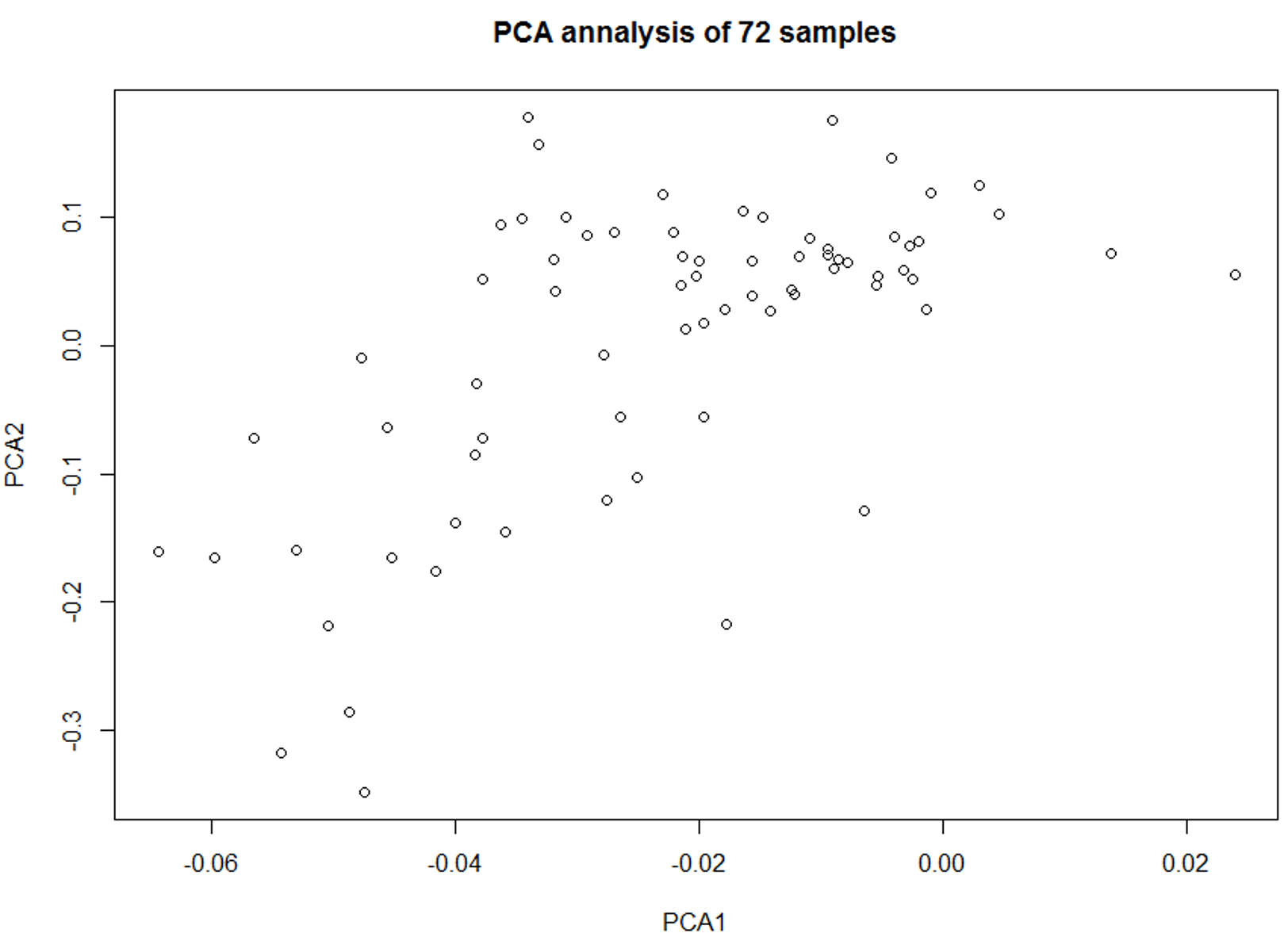

Supplement: Supplementary file 2 — Additional file 2: Fig. S2. Principal Component Analysis of 72 samples. [file 12920_2021_981_MOESM2_ESM.tif]

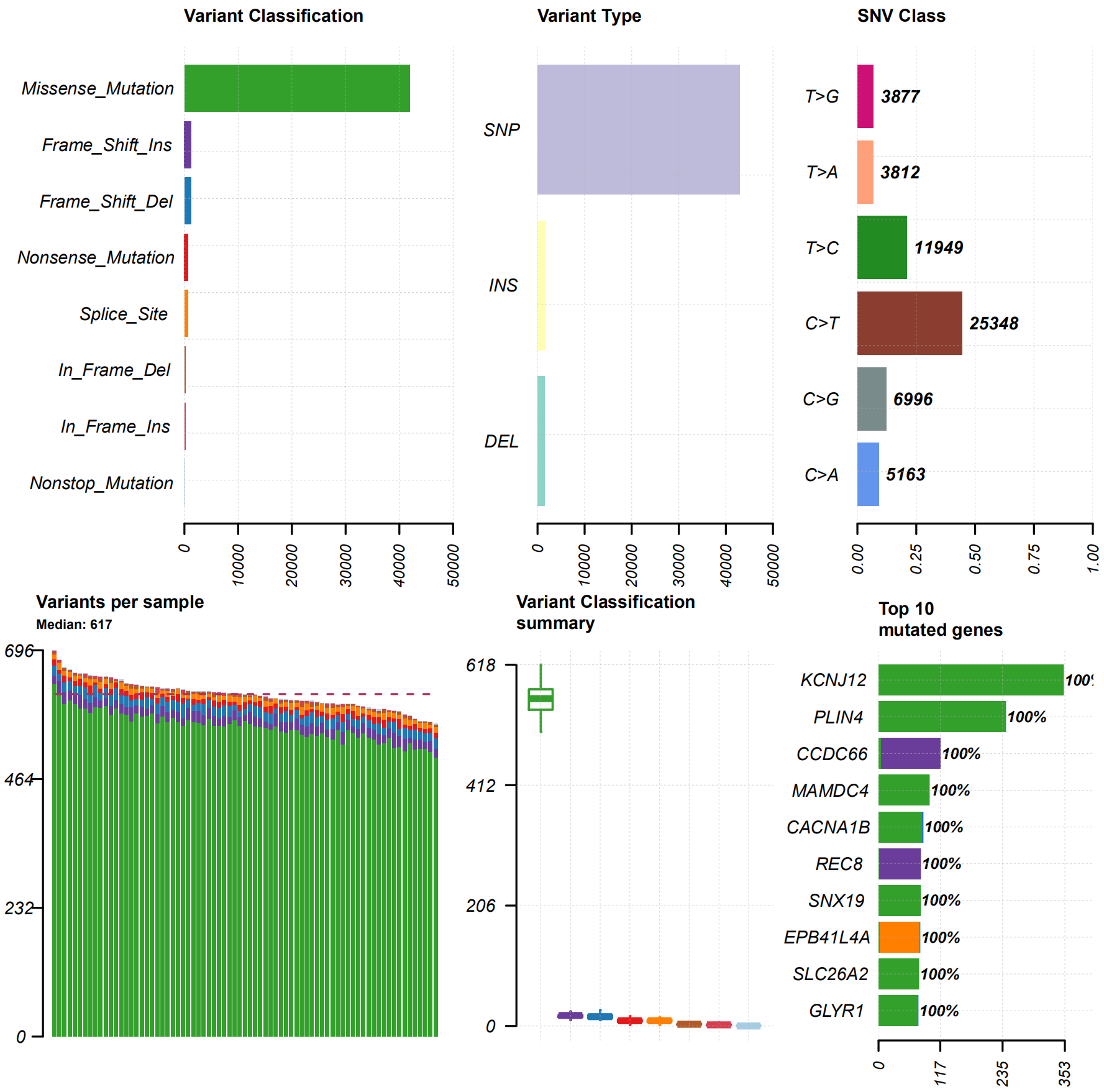

Supplement: Supplementary file 3 — Additional file 3: Fig. S3. Basic variant status in the 75 cases. [file 12920_2021_981_MOESM3_ESM.tif]

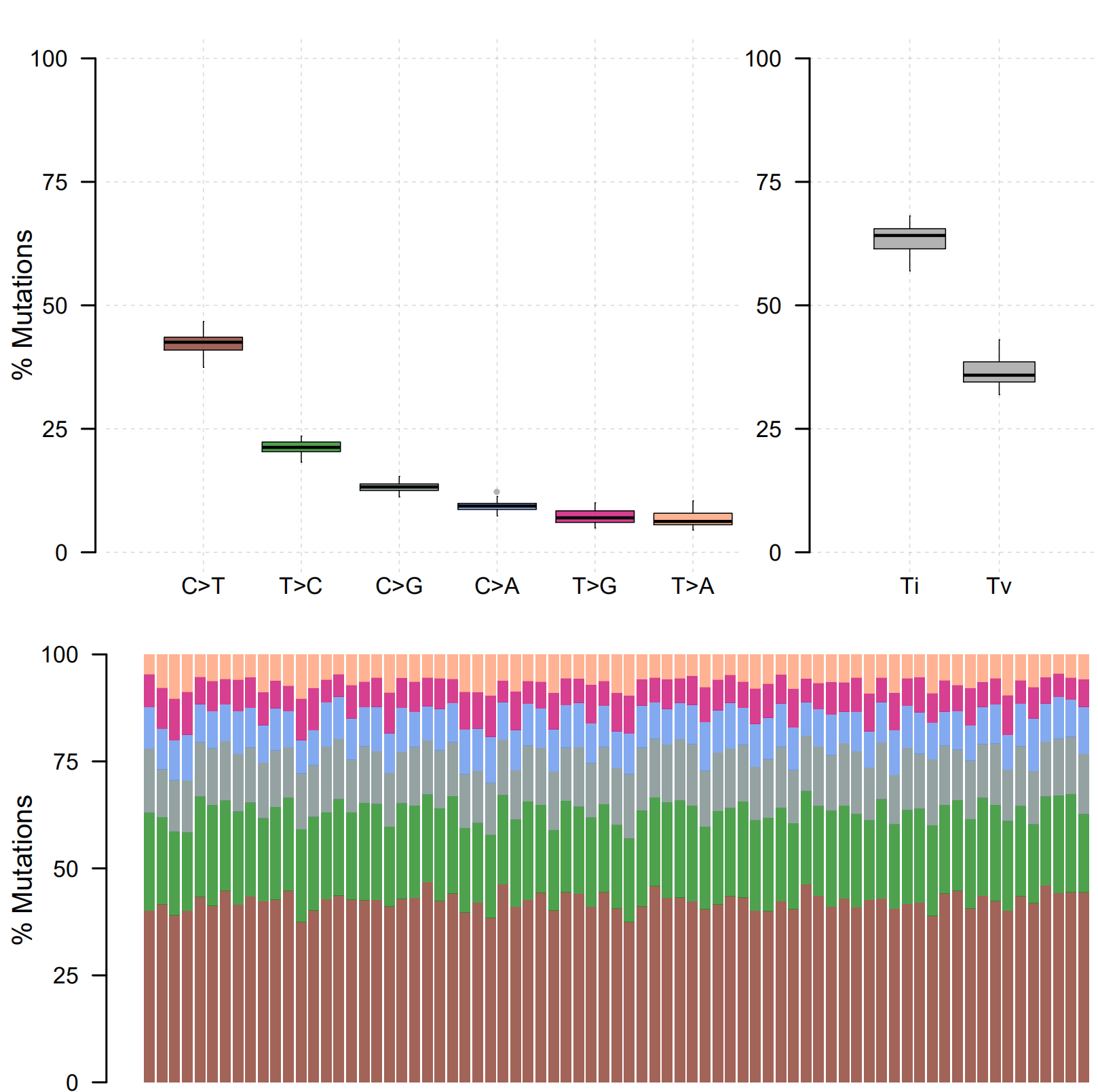

Supplement: Supplementary file 4 — Additional file 4: Fig. S4. Basic variant status in the 75 cases. [file 12920_2021_981_MOESM4_ESM.tif]

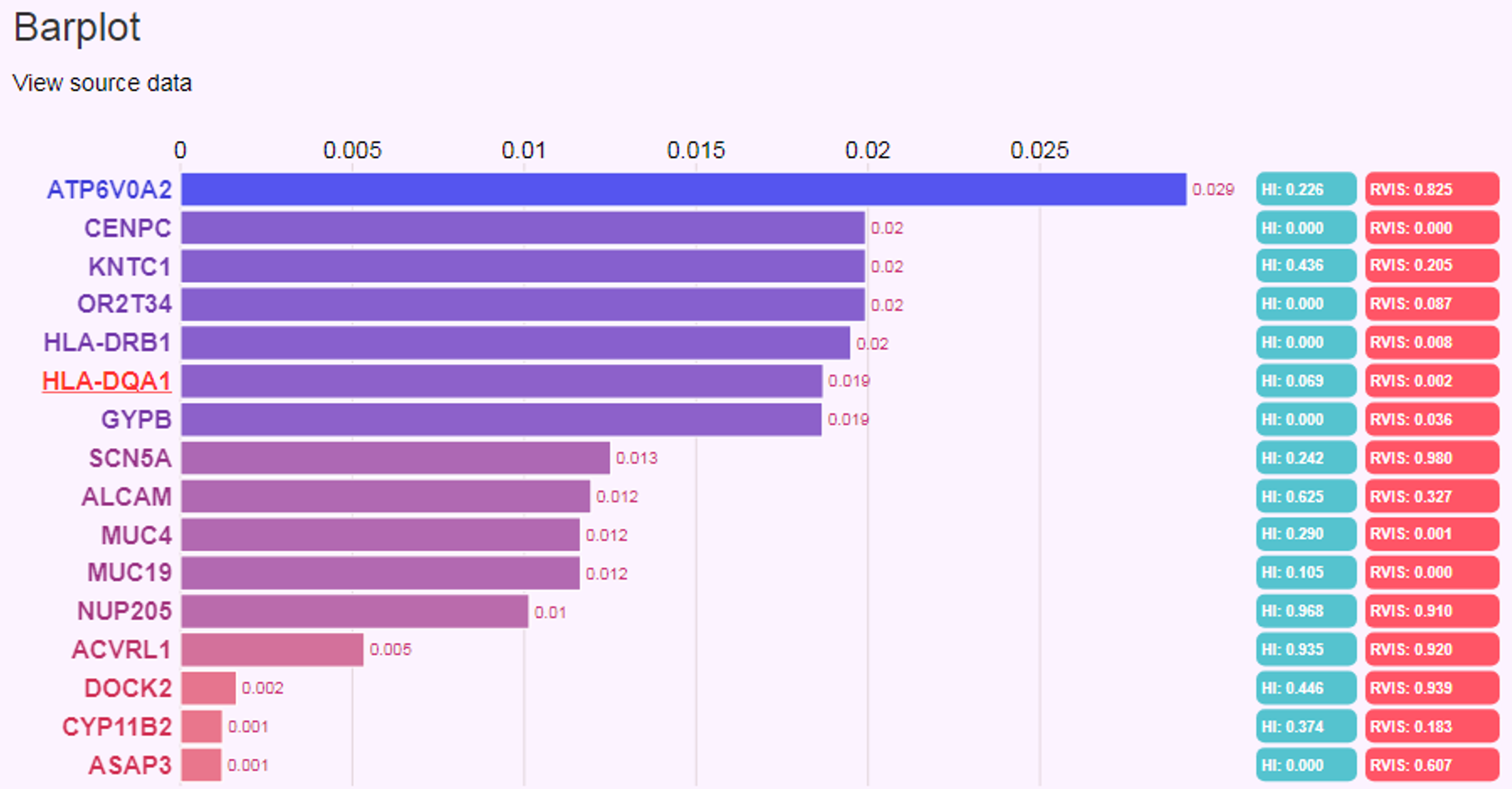

Supplement: Supplementary file 5 — Additional file 5: Fig. S5. Forty-three genes identified by Phenolyzer analysis (Phenolyzer score ≥ 0.01). [file 12920_2021_981_MOESM5_ESM.tif]

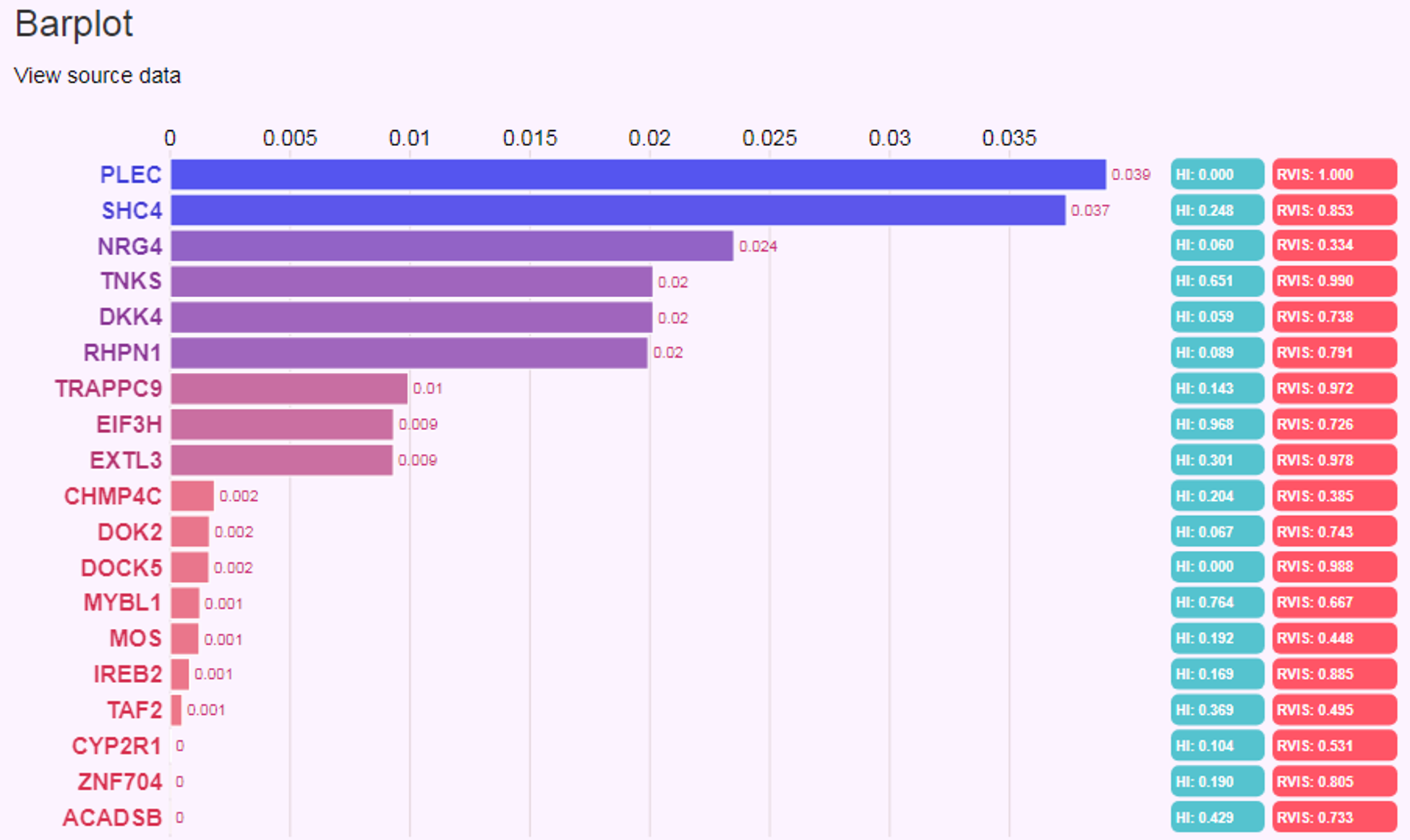

Supplement: Supplementary file 6 — Additional file 6: Fig. S6. Sixty genes identified by Phenolyzer analysis (Phenolyzer score ≥ 0.01). [file 12920_2021_981_MOESM6_ESM.tif]
