# Supplementary material for: The identification of novel gene mutations for degenerative lumbar spinal stenosis using whole-exome sequencing in a Chinese cohort
Source: BMC Med Genomics. 2021 May 21;14:134. doi: 10.1186/s12920-021-00981-4 (PMC8138972; doi:10.1186/s12920-021-00981-4)
Supplement: Supplementary file 7 — Additional file 7: Table. S1. Pathways enriched possibly related to DLSS. [file 12920_2021_981_MOESM7_ESM.docx]

**Supplementary Table 1.** Pathways enriched possibly related to DLSS

| **Pathway** | **Pathway ID** | **Gene** |
| --- | --- | --- |
| Pattern recognition receptors | hsa04054 | *CD207* |
| CD molecules | hsa04090 | *CD207* |
| Lectins | hsa04091 | *CD207* |
| Cytoskeleton proteins | hsa04812 | *FLG* |
| Membrane trafficking | hsa04131 | *GOLIM4* |
| Calcium signaling pathway | hsa04020 | *P2RX5* |
| Neuroactive ligand-receptor interaction | hsa04080 | *P2RX5* |
| Ion channels | hsa04040 | *P2RX5* |
| Membrane trafficking | hsa04131 | *MUC3A* |
| Membrane trafficking | hsa04131 | *MYOT* |
| Cytoskeleton proteins | hsa04812 | *MYOT* |
| Chromosome and associated proteins | hsa03036 | *PDE4DIP* |
| Membrane trafficking | hsa04131 | *SYT15* |
| Toll-like receptor signaling pathway | hsa04620 | *IRF5* |
| Transcription factors | hsa03000 | *IRF5* |
| Osteoclast differentiation | hsa04380 | *SIRPB1* |
| Cell adhesion molecules | hsa04515 | *SIRPB1* |
| CD molecules | hsa04090 | *SIRPB1* |
| Cell adhesion molecules (CAMs) | hsa04514 | *HLA-DQA1* |
| Phagosome | hsa04145 | *HLA-DQA1* |
| Hematopoietic cell lineage | hsa04640 | *HLA-DQA1* |
| Antigen processing and presentation | hsa04612 | *HLA-DQA1* |
| Th1 and Th2 cell differentiation | hsa04658 | *HLA-DQA1* |
| Th17 cell differentiation | hsa04659 | *HLA-DQA1* |
| Intestinal immune network for IgA production | hsa04672 | *HLA-DQA1* |
| Asthma | hsa05310 | *HLA-DQA1* |
| Systemic lupus erythematosus | hsa05322 | *HLA-DQA1* |
| Rheumatoid arthritis | hsa05323 | *HLA-DQA1* |
| Autoimmune thyroid disease | hsa05320 | *HLA-DQA1* |
| Inflammatory bowel disease (IBD) | hsa05321 | *HLA-DQA1* |
| Allograft rejection | hsa05330 | *HLA-DQA1* |
| Graft-versus-host disease | hsa05332 | *HLA-DQA1* |
| Viral myocarditis | hsa05416 | *HLA-DQA1* |
| Type I diabetes mellitus | hsa04940 | *HLA-DQA1* |
| Staphylococcus aureus infection | hsa05150 | *HLA-DQA1* |
| Tuberculosis | hsa05152 | *HLA-DQA1* |
| Human T-cell leukemia virus 1 infection | hsa05166 | *HLA-DQA1* |
| Influenza A | hsa05164 | *HLA-DQA1* |
| Herpes simplex virus 1 infection | hsa05168 | *HLA-DQA1* |
| Epstein-Barr virus infection | hsa05169 | *HLA-DQA1* |
| Toxoplasmosis | hsa05145 | *HLA-DQA1* |
| Leishmaniasis | hsa05140 | *HLA-DQA1* |
| Exosome | hsa04147 | *HLA-DQA1* |
| RNA transport | hsa03013 | *PABPC1* |
| mRNA surveillance pathway | hsa03015 | *PABPC1* |
| RNA degradation | hsa03018 | *PABPC1* |
| Messenger RNA biogenesis | hsa03019 | *PABPC1* |
| Spliceosome | hsa03041 | *PABPC1* |
| Membrane trafficking | hsa04131 | *MUC6* |
| Cytoskeleton proteins | hsa04812 | *DST* |
| Herpes simplex virus 1 infection | hsa05168 | *ZNF468* |
| Transcription factors | hsa03000 | *ZNF468* |
| Membrane trafficking | hsa04131 | *MUC3A* |
| Peptidases and inhibitors | hsa01002 | *AADAC* |
| Thermogenesis | hsa04714 | *SMARCD2* |
| Hepatocellular carcinoma | hsa05225 | *SMARCD2* |
| Transcription machinery | hsa03021 | *SMARCD2* |
| Chromosome and associated proteins | hsa03036 | *SMARCD2* |
| Membrane trafficking | hsa04131 | *MUC21* |
| Cytoskeleton proteins | hsa04812 | *PLEC* |
